# Comparative study of CT-scan modality with MRI modality findings in patients suspected of COVID-19

**DOI:** 10.1186/s43055-023-01009-w

**Published:** 2023-04-26

**Authors:** Mohammad Sobhan Ardekani, Maryam Redaei, Samaneh Ghasemipour, Zahra Ameri Ahmad

**Affiliations:** 1 Shahid Rahnamoun Hospital, Shahid Sadooghi University of Medical Sciences, Yazd, Iran; 2 Chronic Respiratory Disease Research Center, National Research Institute of Tuberculosis and Lung Diseases (NRITLD), Masih Daneshvari Hospital, Shahid Beheshti University of Medical Sciences, Darabad, Tehran, Iran

**Keywords:** COVID-19 pneumonia, Coronavirus, Magnetic resonance imaging, CT-scan

## Abstract

**Background:**

CT-scan and MRI are both best of radiologic modalities with different advantages and disadvantages. In this study, we aimed to evaluate and compare the features of COVID-19 pneumonia in these two modalities. Fifty-three suspected COVID-19 patients who presented to our emergency ward underwent chest CT and, once various features of COVID-19 pneumonia were identified, a dedicated multi-sequence chest MRI was performed on the same day with an institutional protocol. Demographic data and the morphology, laterality and location of the lesions were recorded for each case.

**Results:**

Thirty-seven males and sixteen females with the mean age of 47.49 ± 13.86 years old were present in this case series. Fifty-one cases had typical CT features with ground glass opacities and consolidations, readily visible on different MRI sequences. Thirteen cases had atelectasis which were also easily seen on MRI. The comprehensive review of MRI features for each case and representative images has been illustrated.

**Conclusion:**

We can suggest MRI as an alternative choice of CT-scan for diagnosis COVID-19 pneumonia according to the revealed results, it can be a logical choice in the suspected cases.

## Background

COVID-19 was first reported in the Chinese city of Wuhan and has been pandemic for several months, causing huge financial and human losses and is currently somehow under control with thanks to worldwide vaccination after two years of pandemic. Since the first days of disease report, two methods of chest CT-scan and RT-PCR were used to diagnose COVID-19. Despite concerns over poor specificity and undue radiation exposure, chest CT-SCAN with its high sensitivity in the diagnosis of COVID-19 nonetheless remains central to the initial diagnosis and monitoring of COVID-19 progression, as well as to the evaluation of complications [1]. Although CT-scan has a limitation in differentiation Covid-19 from other viral infections and has a low specificity and also false negative RT-PCR result cannot rules out COVID-19 [2].

Classic features on chest CT include ground glass and reticular opacities with or without superimposed consolidations, frequently presenting in a bilateral, peripheral, and posterior distribution [1]. One of the most important reasons for not controlling the COVID-19 pandemic was the inadequate diagnosis of patients with this highly contagious disease [3]. On the other hand, finding new methods of diagnosis of COVID-19 can help in more controlling this disease and also finding new methods of staging for COVID-19 can affect the prognosis by choosing the most appropriate treatment method.

Although thoracic MRI is not routinely effective in diagnosing or staging COVID-19 severity, studies have shown that MRI can be used to diagnose and stage COVID-19 severity. More recently, studies conducted with MRI have shown excellent concordance with chest CT in visualizing typical features of COVID-19 pneumonia. For patients in whom exposure to ionizing radiation should be avoided, particularly pregnant patients and children, pulmonary MRI may represent a suitable alternative to chest CT. For example, a study published by Deen et al. showed that pulmonary lesions due to COVID-19 appeared as high T2 and T1 signals and high DWI signals [2]. The aim of this study was to compare the findings of MRI and chest CT-SCAN modality in patients with COVID-19.

## Methods

### Study design

This cross-sectional study was approved by the Ethics Committee (Approval No.: IR.SSU.Medicine.Rec.1400.389) for the Protection of Human Subjects of Yazd University of Medical Sciences. The costs of this research project were funded by the Research Committee of Yazd University of Medical Sciences and the Vice-Chancellor of Research.

### Study population

This study included 53 cases suspected of COVID-19 referred to Shahid Rahnemoun Hospital in Yazd and underwent Chest CT-scan (HRCT). All the included patients CT-scan imaging confirmed COVID-19 disease in addition to clinical findings. It is necessary to be mentioned that all the included patients were stable in the subject of respiratory, cardiac and medical status. All of the study steps were clarified for each patient prior to the engagement in the study. Age of patients ranged from 23 to 77 years; the average age was 47 ± 13 years (Table 1.)

**Table 1:** Determining the frequency of patients by gender

Gender	Number	Percent
Male	37	69.8
Female	16	30.2
Total patients	53	100

### CT-scan and MRI

CT-scan of patients was performed by Siemens/16 slice brand with HRCT protocol and lung window view with head first position. The HRCT protocol in this study included: 100 k/v, slice thickness 5 mm, space between slices 1.5 mm, lung construction filter. The average effective dose was 40 mSv. Patients were referred for MRI after CT-SCAN with informed consent (at the same day). MRI was done with: 120 kV, with adaptive tube current modulation, exposure time 400 ms, slice thickness 1.25 mm, spaces between slices 0.5 mm, lung reconstruction filter. The average effective dose was 2 mSv.

MRI of the study was conducted on 3 T scanner (AVENTO 1/5 TESLA) in the supine position using the abdominal and spinal radiofrequency coils. MRI protocol included: Axial T2 Haste (T2 half Fourier single shot turbo spin echo), Sagittal: T2 Haste, Transverse: T2 Haste fat suppression; T2 true FISP. MRI protocol was conducted based on TR: 800, TE: 350, Flip angle: 150, for phase: 100%, slice thickness: 4.5 mm. To minimize dynamic artifacts associated with respiratory movements, single shot with respiratory gating was used. All the protocols were implemented for each patient about a total cycle time of 12–15 min.

It is good and also necessary to be mentioned that all the safety measures were in accordance with the prior WHO guidelines for COVID-19.

During the examination of patients suspected or confirmed for COVID-19, all safety measures were taken, namely: all department personnel was using personal protective equipment and FFP2 respirators. Patients were wearing a surgical mask. In addition, all parts of the scanner that came into contact with patients were covered by disposable covers, which were disposed after each patient immediately and also disinfected with hospital-designed medical equipment’s sanitizer. After the completion of the study, the scanner's surfaces were disinfected.

### Radiologic assessment

All the information of patients radiologic images (CT-scan and MRI) was studied and analyzed by 2 experienced accurate radiologist with more than 10 years of clinical and academic experience. Diagnostic criteria for reporting CT-scans as positive of COVID-19 were as follows: ground glass opacity (peripheral or diffused), consolidations, atelectasis, crazy paving, septal thickening, etc.

All the findings of the CT-scan were classified reported according to the European Society of radiology-European Society of thoracic imaging (ESR-ESIT) in five grades as follows; grade I: less than 10 percent; grade II: 25 percent, grade III: 25–50 percent, grade IV: 51–74 percent and grade V: more than 75 percent.

During preparation of reports of the study radiology findings, we paid a very accurate attention to the polysegmented sections with isointense signal which were reflecting GGO. Images were also evaluated for the presence of areas of homogeneous hyper-intense signal corresponding to pulmonary consolidation on CT-scans, as well as for ‘crazy-paving’ sign corresponding to combination of GGO and pronounced thickening of interlobular septa.

Reviewing of CT-scans was performed by two radiologists who were not aware of the results according to the researcher-designed checklist. CT-scan of patients was performed and then followed up for MRI with informed consent. It should be mentioned that radiologists were not aware of MRI results also.

### Statistical analysis

Statistical analysis was performed to compare the findings of two radiology modalities (CT-SCAN and MRI) in suspected patients of COVID-19. A proportion of patients with the presence of lesions on T2WI and on DWI was compared according to the McNamara criteria. The significance level for all criteria is set to *P* < 0.05.

## Results

In this study, pulmonary involvement of 47 patients (88.7%) was reported as diffuse and pulmonary involvement of 6 patients (11.3%) as peripheral (Table 2).

**Table 2:** Determining the type of lung involvement of the studied patients

Type of lung involvement	Number	Percent
Diffuse	47	88.7
Peripheral	6	11.3
All of patients	53	100

Study of pathological findings related to CT-scan of patients was as follows: GGO in 51 patients (96.2%), consolidation in 32 patients (60.4%), atelectasis in 13 patients (24.5%), fibrosis in 2 patients (3.8%) and pulmonary nodule in 1 patient (1.9%) reported. Study of pathological findings related to MRI of patients was as follows: GGO in 51 patients (96.2%), consolidation in 32 patients (60.4%), atelectasis in 11 patients (20.8%) and fibrosis in 2 patients (3.8%). There was a difference between CT-scan and MRI only in the number of reported atelectasis, but McNamara test showed that this difference was not significant (*P* > 0.05). Lymphadenopathy, pleural effusion, emphysema, linear opacity, reverse halo sign, cavitation, crazy paving, peri-bronchial thickening, and mosaic attenuation were not reported in any patient (Table 3).

**Table 3:** Comparison of pathological findings of CT-scan and MRI of patients

Pathologic findings	CT-scan				MRI				*P* value
	Has		Does not have		Has		Does not have		
	Number	Percent	Number	Percent	Number	Percent	Number	Percent	
Consolidation	32	60.4	21	39.6	32	60.4	21	39.6	1
Fibrosis	2	3.8	51	96.2	2	3.8	51	96.2	1
Pulmonary nodule	1	1.9	52	98.1	–	–	–	–	–
Atelectasis	13	24.5	40	75.5	11	20.8	42	79.2	0.68
Ground glass opacity (GGO)	51	96.2	2	3.8	51	96.2	2	3.8	1
Linear opacity	–	–	–	–	–	–	–	–	–
Reserve halo sign	–	–	–	–	–	–	–	–	–
Emphysema	–	–	–	–	–	–	–	–	–
Peribronchial thickening	–	–	–	–	–	–	–	–	–
Pleural effusion	–	–	–	–	–	–	–	–	–
Mosaic attenuation	–	–	–	–	–	–	–	–	–
LAPCT	–	–	–	–	–	–	–	–	–
Crazy paving	–	–	–	–	–	–	–	–	–

The indicators of the diagnostic value of MRI compared to CT-scan for the pathological findings of suspected COVID-19 patients were obtained as follows:

The sensitivity and specificity of MRI for the diagnosis of Consolidation, GGO and fibrosis were 100%, and the positive and negative predictive value of MRI for the diagnosis of these 3 findings was 100%.

The sensitivity of MRI for diagnosing atelectasis was 84.6% and its specificity was 100%; also, the positive predictive value of MRI for diagnosing atelectasis was 100% and its negative predictive value was 95.2%.

Considering that the number of pulmonary nodules reported in all patients was only one case that was reported only in the CT-scan of the patient, it was not possible to calculate the sensitivity and positive predictive value of MRI for the diagnosis of pulmonary nodules, but the specificity and negative predictive value of MRI for the diagnosis of pulmonary nodule in patients were 100% and 98.1%, respectively.

Pulmonary involvement in CT-scan of patients was less than 10% in 5 patients (9.4%), between 10 and 25% in 19 patients (25.8%), between 25 and 50% in 17 patients (32.1%), between 50 and 75% in 11 patients (20.8%) and more than 75% in 1 patient (1.9%).

Pulmonary involvement in MRI of patients was less than 10% in 12 patients (22.6%), between 10 and 25% in 13 patients (24.5%), between 25 and 50% in 20 patients (37.7%), between 50 and 75% in 6 patients (11.3%) and more than 75% in 2 patients (3.8%).

Chi-square test showed that the difference in reporting the percentage of pulmonary involvement between CT and MRI modalities was significant (*P* < 0.05) (Table 4).

**Table 4:** Comparison of the percentage of lung involvement in CT-scan and MRI of patients

Variable (%)	CT-scan		MRI		*P* value
	Number	Percent	Number	Percent	
*Percent of lung involvement*					
< 10	5	9.4	12	22.6	< 0.0001
10–25	19	35.8	13	24.5	
25–50	17	32.1	20	37.7	
50–75	11	20.8	6	11.3	
> 75	1	1.9	2	3.8	

The sensitivity of MRI in determining the involvement of less than 10% of the lung was 100% and its specificity was 85.4%; also, the positive predictive value was 41.6% and the negative predictive value was 100%. The sensitivity of MRI in determining the involvement of 10–25% of the lung was 68.4% and its specificity was 100%; also, the positive predictive value was 100% and the negative predictive value was 85%.

The sensitivity of MRI in determining the involvement of 25–50% of the lung was 100% and its specificity was 91.6%; also, the positive predictive value was 85% and the negative predictive value was 100%. The sensitivity of MRI in determining involvement of more than 50% of the lung was 66.6% and its specificity was 100%; also, the positive predictive value was 100% and the negative predictive value was 91.1%.

The size of lesions in CT-scan of patients was reported as less than 1 cm in 16 patients (30.2%), between 1 and 3 cm in 25 patients (47.2%) and more than 3 cm in 12 patients (22.6%).

The size of lesions in MRI of patients was reported as less than 1 cm in 15 patients (28.3%), between 1 and 3 cm in 29 patients (54.7%) and more than 3 cm in 9 patients (17%).

Chi-square test showed that the difference in the report of lung lesion size between the two modalities of CT and MRI was significant (*P* < 0.05) (Table 5).

**Table 5:** Comparison of lesion size in CT-scan and MRI

Variable	CT-scan		MRI		*P* value
	Number	Percent	Number	Percent	
*Size of lesions*					
< 1 cm	16	30.2	15	28.3	< 0.0001
1–3 cm	25	47.2	29	54.7	
> 3 cm	12	22.6	9	17	

The diagnostic value indicators of MRI compared to CT-scan to determine the size of lung lesions in suspected patients with COVID-19 were obtained as follows:

The sensitivity of MRI in determining the size of lesions less than 1 cm was 93.7% and its specificity was 100%; also, the positive predictive value was 100% and the negative predictive value was 97.3%.

The sensitivity of MRI in determining the size of lesions from 1 to 3 cm was 100% and its specificity was 85.7%; also, the positive predictive value was 86.2% and the negative predictive value was 100%.

The sensitivity of MRI in determining the size of lesions greater than 3 cm was 75% and its specificity was 100%; also, the positive predictive value was 100% and the negative predictive value was 93.1%.

**Table:** 

Case no. 1	
Chief complaint: 32 years old male with fever, tachypnea and dyspnea	
Chest CT-scan	Chest MRI
Findings: ground glass opacity at upper segment of right lower lobe	Findings: ground glass like signal-abnormality (hyper-signal) on all sequences
	
	

**Table:** 

Case no. 2	
Chief complaint: 32 years old male with mild dyspnea	
Chest CT-scan	Chest MRI
Findings: faint ground glass pattern density in posterior segment of right upper lobe	Findings: minimal signal abnormality (hyper-signal) on all sequences in posterior segment of right upper lobe
	
	

**Table:** 

Case no. 3	
Chief complaint: 60 years old female with dyspnea and fever	
Chest CT-scan	Chest MRI
Findings: consolidation in lateral segment of right middle lobe and upper segment of right lower lobe	Findings: consolidation like hyper-signal changes on all sequences in lateral segment of right middle lobe and upper segment of right lower lobe
	
	

**Table:** 

Case no. 4	
Chief complaint: 63 y/o male with fever and tachypnea and dyspnea	
Chest CT-scan	Chest MRI
Findings: ground glass density in lateral segment of right middle lobe(tree in bud pattern due to active viral infection), Patchy ground glass density in periphery of left upper lobe	Findings: ground glass like hyper-signal changes on all sequences at lateral segment of right middle lobe, Patchy ground glass like signal abnormality in periphery of left upper lobe
	
	

**Table:** 

Case no. 5	
Chief complaint: 53 y/o male with fever and tachypnea and dyspnea	
Chest CT-scan	Chest MRI
Findings: patchy consolidation in right middle lobe (medial and lateral segment)	Findings: patchy consolidation like hyper-signal changes on all sequences in right middle lobe (medial and lateral segment)
	
	

**Table:** 

Case no. 6	
Chief complaint: 52 years old male with fever and tachypnea and dyspnea	
Chest CT-scan	Chest MRI
Findings: multiple patchy ground glass density in both lungs	Findings: multiple patchy ground glass like hyper-signal abnormality on all sequences in both lungs
	
	

## Discussion

Although most patients with the novel coronavirus disease had mild to moderate symptoms with good prognosis, in some cases, it was complicated by severe damage to the respiratory system [5]. CT-scan is the most sensitive among all imaging modalities for detection of changes in pulmonary parenchyma. According to the international expert consensus, CT has become the method of choice for patients with suspected viral pneumonia, since its results directly affect patient management [6]. Although American College of Radiology statement recommends minimizing MRI utilization in COVID-19 pandemic, urgent cases are still performed, and elective MRIs will show increasing trend in upcoming days [7].

It has been shown that lung MRI is as efficient and accurate as chest CT imaging in providing fine details of the lung parenchyma and pleural abnormalities in patients with lower respiratory tract infection [8], but the actual practical role is limited by loss of signal due to physiologic respiratory and cardiac motion, and low amount of hydrogen protons in the lung parenchyma [9]. Hence, various radiologic manifestations of COVID-19 pneumonia such as GGO, consolidations and ill-defined reticulations can be easily distinguished with lung MRI.

This article presents a comparative information with an acceptable number of participants with an approach to provide highly informative chest MRI as the alternative for chest CT in patients with COVID-19, especially in cases with high risk for CT imaging. There are a variety of studies worked on clinical cases corresponding CT and MRI comparison [2–4, 10–12].

In a study aimed at evaluating the findings of thoracic CT-scan in 51 patients with COVID-19 revealed that the most common findings include lesions of ground glass opacity, consolidation and increase in inter-lobar thickness. However, none of these findings are specific for COVID-19 and do not help differentiate COVID-19 from other viral pneumonias and our study results revealed that GGO, consolidation and atelectasis were the most lesions in CT-scan and MRI modality [2].

CT and MRI most common finding was GGOs in 14 and 16 study cases, respectively. One of the cases underwent CT-scan 3 days after MRI and revealed GGO at the same region previously diagnosed by MRI, indicating acceptable sensitivity of MRI compared to CT-scan in detecting GGO [10].

In a case–control study by Deen et al. showed that COVID-19-induced lung lesions in MRI modality appear as high T2 and T1 signal and high DWI signal. In this study, the patient undergoes liver MRI, which in MRI shows the lower parts of the lung as high signal lesions at T1 and T2 and the diagnosis of COVID-19 is then confirmed by RT-PCR which is consistent with the findings of our study [3].

Yang et al. in a prospective clinical trial on 23 patients with COVID-19 who underwent MRI showed that MRI of the lung is highly consistent with the findings of the CT-scan modality of the lung and is as efficient as CT-scan of the lung for evaluating patients with COVID-19. This trial results is the same as our results for considering MRI imaging modality as effective as CT-scan and even safer alternative [11].

Along with our results, Ekinci et al. study revealed similar findings: Almost all the MRI sequences were consistent with detected locations of consolidations on CT-scans images. They reported that MRI can be used as an alternative to CT-scan in cases dynamic monitoring of patients is required in order to avoid ionizing radiation exposure [12].

The main question prior to our study mentioned by a study [4] was to what degree detected findings on MRI scans of COVID-19 patients correlate with the radiological changes detected by CT-scan. Our study compared the picture of pathological changes in the lungs obtained with CT and MRI at least in small samples of patients with SARS-CoV-2 pneumonia and showed the sensitivity of MRI in detecting the same lesions compared to CT.

Overall, our study showed that chest MRI can act as a potential alternative to chest CT in diagnosis of COVID-19 pneumonia especially in high risked cases whom CT-scan is contraindicated for them, although further studies are warranted. One of the major limitations of utilizing MRI in contagious disease such as COVID-19 is the potential risks of infection control after using the imaging instrument. On the other hand, the strength point of our study is the population with an acceptable different spectrum ages of 23–77 years.

The main strength of our study is an acceptable sample size of 50, which allow us to make confident conclusions, as well as comparing the diagnostic findings corresponding COVID-19 on MRI and CT images of the same patient.

## Conclusion

As we know, thorax and specifically chest imaging still continues to remain essential to the monitoring and staging of COVID-19 pneumonia. Since from the beginning of COVID-19 outbreak, chest CT-scan was announced as the method of choice for diagnosis, getting familiar with MRI routinely is somehow time taking for physicians. Although chest MRI is not mentioned among first-line diagnostic radiologic modalities specifically in detecting and differentiating pulmonary COVID-19, but our study along with other researches results showed the same capability of it comparing to CT-scan. According to the results of our research, MRI has the same sensitivity as CT-Scan in detection and locating COVID-19 pneumonia, especially in high risked and CT-contraindicated cases.

We can suggest MRI as an alternative choice of CT-scan for diagnosis COVID-19 pneumonia according to the revealed results. For patient groups in whom excessive or repeated exposure to ionizing radiation should be avoided, pulmonary MRI may yet provide a viable alternative.

It is worth to be mentioned that one of the most advantages of MRI compared to CT-scan is the ability of monitoring disease dynamics.

## Recommendations for further research

Concerning the percentage of pulmonary involvement, MRI has significantly showed downgraded percentage of pulmonary involvement, so this could be considered as a point of view for evaluation for the further studies. Also, there was a significant difference in the size of the affected area when measured by CT and MRI which these findings could be studied as a systematic review with a considerable sample size to check the accuracy of these two imaging modalities more precisely.

## Data Availability

Data and material statement are available and reachable for who may ask for. The datasets used and/or analyzed during the current study are available from the corresponding author on reasonable request.

## Abbreviations

CT-scan: Computed tomography scan
MRI: Magnetic resonance imaging
COVID-19: Coronavirus disease 2019
RT-PCR: Real-time polymerase chain reaction
HRCT: High-resolution computed tomography
WHO: World Health Organization
FFP-2: Filtering FacePiece-2
GGO: Ground glass opacity
